# Investigation on the Material Removal and Surface Generation of a Single Crystal SiC Wafer by Ultrasonic Chemical Mechanical Polishing Combined with Ultrasonic Lapping

**DOI:** 10.3390/ma11102022

**Published:** 2018-10-18

**Authors:** Yong Hu, Dong Shi, Ye Hu, Hongwei Zhao, Xingdong Sun

**Affiliations:** School of Mechanical and Aerospace Engineering, Jilin University, 5988 Renmin Street, Changchun 130025, China; huyong@jlu.edu.cn (Y.H.); shidong16@mails.jlu.edu.cn (D.S.); self-song@163.com (Y.H.); xdsun15@mails.jlu.edu.cn (X.S.)

**Keywords:** single crystal SiC, material removal, surface generation, ultrasonic chemical mechanical polishing, ultrasonic lapping

## Abstract

A new method of ultrasonic chemical mechanical polishing (CMP) combined with ultrasonic lapping is introduced to improve the machining performance of carbide silicon (SiC). To fulfill the method, an ultrasonic assisted machining apparatus is designed and manufactured. Comparative experiments with and without ultrasonic assisted vibration are conducted. According to the experimental results, the material removal rate (MRR) and surface generation are investigated. The results show that both ultrasonic lapping and ultrasonic CMP can decrease the two-body abrasion and reduce the peak-to-valley (PV) value of surface roughness, the effect of ultrasonic in lapping can contribute to the higher MRR and better surface quality for the following CMP. The ultrasonic assisted vibration in CMP can promote the chemical reaction, increase the MRR and improve the surface quality. The combined ultrasonic CMP with ultrasonic lapping achieved the highest MRR of 1.057 μm/h and lowest PV value of 0.474 μm. Therefore this sequent ultrasonic assisted processing method can be used to improve the material removal rate and surface roughness for the single crystal SiC wafer.

## 1. Introduction

Being one of the third-generation (wide bandgap) semiconductor materials, carbide silicon (SiC) has attracted much attention both in academic research and industrial application, because it has remarkable physical and chemical properties such as high thermal conductivity, high hardness, chemical resistance, temperature resistance, transparency to light wave, etc. [1]. Thus, SiC has become one of the most promising materials in optoelectronics and power electronics, especially when the device is used in a high temperature, high frequency, high power, and radiation resistant environment. However, its mechanical and chemical properties make the surface planarization much more difficult than silicon and sapphire, which are extensively used as substrate materials, as its Mohs hardness is close to diamond and the chemical inertness is too strong to react in all known aqueous etching solutions at room temperature except in hot Hydrofluoric Acid (HF) solution or phosphoric acid above 200 °C [2]. It is well known that chemical mechanical polishing (CMP) is still the most efficient technology to finish the hard-brittle wafer, which can achieve the ultra-smooth surface with minimal damage [3]. In addition, CMP of SiC substrate using colloidal silica slurry was first reported in 1997 by Zhou et al. [4]. This research reported that high temperature and a pH higher than 10 were indispensable conditions to remove material. But the material removal rate (MRR) was still very low, typically less than 100 nm/h.

On considering improving the material removal rate (MRR) of SiC during CMP, the reported literature mainly focuses on CMP slurry [5,6,7,8,9,10]. On the one hand, CARE (catalyst-referred etching) [11] using different oxidants and catalysts was studied to replace the high temperature. Among the oxidants, hydrogen peroxide (H_2_O_2_) was the most widely used to increase the chemical corroding of SiC [7,9]. In addition, Fe nanoparticles or hydroxyl ion was the most widely used catalyst for H_2_O_2_ [8,10], but the MRR was still not large. For example, the MRR was 60 nm/h when only H_2_O_2_ was added into the slurry. The MRR was 100 nm/h when both H_2_O_2_ and hydroxyl ion were added into the slurry, and it was not more than 200 nm/h when the slurry contained H_2_O_2_ and Fe nanoparticles. On the other hand, the addition of a high-hardness abrasive combined with an oxidizer to the slurry is another method to increase MRR. Heydemann et al. [5] increased the MRR to about 900 nm/h by addition of a nano-diamond and H_2_O_2_. Jeong et al. [6] increased the MRR to about 500 nm/h also by addition of a nano-diamond. But the cost of a nano-diamond abrasive is expensive, and the diamond particle easily produces mechanical scratches. From the view of processing, ECMP (electro-chemical mechanical polishing) [12], PAP (plasma assisted polishing) [13], and PCMP (photocatalysis-assisted CMP) [14] have been reported to increase the MRR of single-crystal SiC. Electro-chemical mechanical polishing is suitable for the conductor and needs an electrical system with a cathode in the slurry. Plasma assisted polishing needs high temperatures, large amounts of power, and complicated equipment, while the MRR is still not large. Photocatalysis-assisted CMP needs a photocatalyst, ultraviolet light, an electron capturer, and an acid environment, and the MRR is still not large enough.

In contrast, ultrasonic assisted CMP has been reported to be a cost-efficient method to improve the processing performance. For example, Tsai et al. [15] combined the ultrasonic CMP with ultrasonic dressing of a diamond disk to increase the MRR for copper substrate with an improved surface roughness and the torque force was dramatically reduced. The research provided a feasible method to improve the polishing performance and dressing efficiency simultaneously. Lu et al. [16,17] increased the MRR, lowered the surface roughness, and improved the surface flatness for a sapphire substrate by virtue of ultrasonic flexural vibration assisted CMP with a self-designed flexural vibrating plate. Li et al. [18,19] made experimental studies on ultrasonic and megasonic vibration assisted CMP for silicon wafers and the results implied a better effect than CMP without ultrasonic. Liu et al. [20] also had a study on the elliptical vibration-aided CMP of monocrystalline silicon, where a mathematical model of MRR and the experimental verification were performed. And a good match between the mathematical model and experimental results was achieved.

However, ultrasonic assisted CMP of single crystal SiC has been hardly reported. Additionally, ultrasonic assisted abrasive machining can decrease the machining forces thereby reducing surface damage [21,22,23]. Thus, this paper introduces the ultrasonic CMP combined with ultrasonic lapping to process the single crystal SiC wafer based on a self-devised and manufactured ultrasonic unit. In addition, the specimens are the Si-face SiC, which are considered to be more useful for epitaxial film growth, but more difficult to be removed compared with C-face [9]. A series of combinational experiments with and without ultrasonic were conducted to investigate the influence of ultrasonic on the MRR and surface generation of SiC, where the surface roughness may not reach the level reported in the references. Firstly, the effect of ultrasonic on the chemical corrosion of Si-face SiC during CMP is investigated; secondly, the effect of ultrasonic on the CMP performance of Si-face SiC is investigated; finally, the surface status is observed by the optical microscope and AFM, the chemical corroding is measured by XPS, and the MRR is calculated according to the mass change measured by electronic balance.

## 2. Experimental Section

### 2.1. Experimental Setup

**Figure 1:**
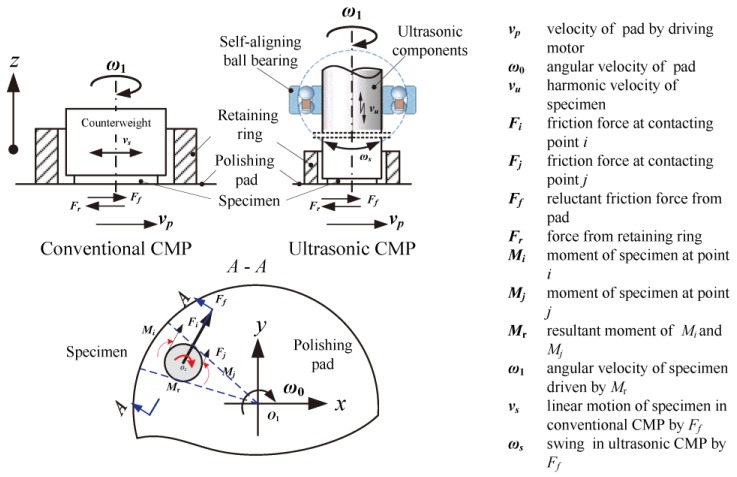
Dynamical schematic diagram of ultrasonic chemical mechanical polishing (CMP) compared with conventional CMP.

The dynamical principle of ultrasonic CMP compared with conventional CMP is shown in Figure 1. The ultrasonic CMP system employs such major functional components as ultrasonic components, self-aligning ball bearing, retaining ring, polishing pad, and specimen. The conventional CMP system employs such major components as a counterweight, retaining ring, polishing pad, and specimen. The ultrasonic components can provide both vertical harmonic vibration (*v*_u_) and adjustable polishing pressure for the specimen while the counterweight in a conventional CMP only provides invariable pressure. The self-aligning ball bearing can rotate in all directions around its symmetric center (*ω*_s_), which can provide a self-adaptive contact for the specimen and polishing pad. The retaining ring is used to prevent the components from being thrown off the polishing pad by the action of centrifugal force. It can also evenly spread the slurry and maintain a thin film layer of slurry between specimen and polishing pad, simultaneously maintaining the flatness of the polishing pad. The specimen rotates around its axial center in the retaining ring, where the rotational movement (*ω*_1_) is driven by the reluctant friction force (*F*_r_) of the polishing pad.

**Figure 2:**
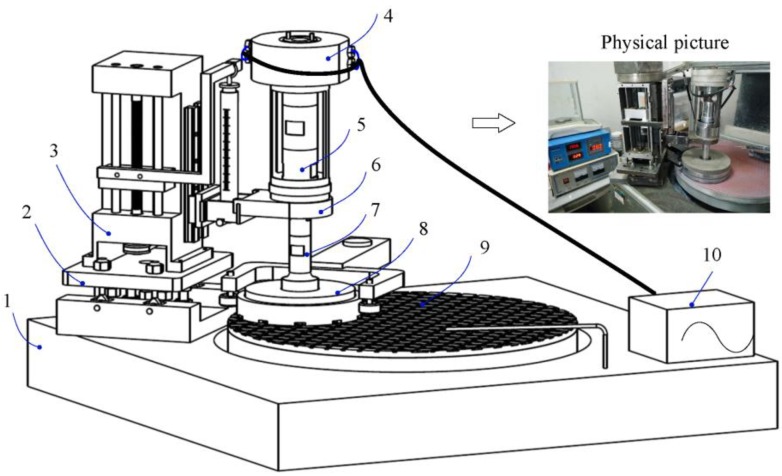
Structural diagram and physical picture of ultrasonic CMP system: (**1**) polisher (Bni62, Japan); (**2**) universal platform; (**3**) load adjuster; (**4**) sliding ring components; (**5**) transducer; (**6**) self-aligning ball bearing; (**7**) stepped horn; (**8**) retaining and dressing ring; (**9**) polishing pad; (**10**) ultrasonic digital power source.

Based on the dynamical principle, the structure compositions of the ultrasonic CMP system for the subsequent ultrasonic assisted experiments were designed and manufactured, which are shown in Figure 2. This experimental setup comprises such major structural components as the commercial polisher (Bni62, BN Technology Corporation, Tokyo, Japan), universal platform, load adjuster, sliding ring components, transducer, self-aligning ball bearing, stepped horn, retaining and dressing ring, polishing pad, and ultrasonic digital power source. The function and assembly relationship of the major components are elaborated as follows. The universal platform can provide all the rotational degrees-of-freedom for the load adjuster and the self-aligning ball bearing can also provide all the rotational degrees-of-freedom for the stepped horn. Therefore, the combinational action of universal platform and self-aligning ball bearing can ensure the good adaptive contact for the specimen and polishing pad. The load adjuster is mainly composed of a ball screw nut kinematic pair, linear guide kinematic pair, belt drive pair, and dynamometer. It is used to provide a controllable polishing pressure for the specimen by changing the spring length of the dynamometer. The transducer is a sandwich structure and produces the ultrasonic vibration through the piezoelectric effect of piezoelectric ceramic. The designed stepped horn is used to change the ultrasonic amplitude of the transducer. The digital power source outputs the ultrasonic signal to the transducer by the sliding ring components. And the specimen is mounted on the top of the stepped horn. The retaining ring is fixed on the dressing ring, which is used to constrain all the degrees-of-freedom of the stepped horn in the horizontal direction and keep the stepped horn rotating around its axial center. 

### 2.2. Design of Experiments and Performance Characterization

In order to investigate the effect of ultrasonic on the chemical corrosion of single crystal SiC during CMP and the effect of ultrasonic on the CMP performance of single crystal SiC, the comparative experiments of conventional and ultrasonic chemical corrosion, conventional and ultrasonic CMP were arranged. Before chemical corrosion and CMP, the specimens were processed after the ultrasonic and conventional lapping separately based on the aforementioned ultrasonic system. The designed combinational experiments for chemical corroding and CMP are both listed in Table 1, which were conventional corroding/CMP after conventional lapping, ultrasonic corroding/CMP after conventional lapping, conventional corroding/CMP after ultrasonic lapping, and ultrasonic corroding/ CMP after ultrasonic lapping. Additionally, the related lapping and CMP parameters with the corresponding values are shown in Table 2 and Table 3, respectively, which can be categorized as ultrasonic vibration parameters, mechanical action parameters, slurry parameters, and processing time. Among the parameters, the value of ultrasonic vibrated amplitude was determined by laser Doppler vibration meter. To evaluate the experimental effect, surface topography, roughness, and material removal rate (MRR) were set as the performance characterization indexes. The surface topography and roughness were observed by atomic force microscopy (AFM, Bruker Dimension Icon, Massachusetts, America) and optical microscope. The MRR was calculated by the Equation (1).

(1) $$MRR=\frac{\Delta h}{t}=\frac{\Delta m/\rho_{\mathrm{sic}}A}{t}$$

where Δ*m* is the weight loss of SiC wafer measured by professional electronic balance (resolution: 0.0001 g), *ρ*_sic_ is the density equaling to 3.21 g/cm^3^, *A* is polishing area, *t* is the polishing time. In addition, X-ray photoelectron spectroscopy (XPS, ThermoFisher Scientific, Massachusetts, America) was used to study the surface corroded layer of single crystal SiC during CMP.

## 3. Results and Discussion

Before measurements of XPS, weight loss, and AFM, the processed SiC required cleaning. According to the theory of “similarity and intermiscibility”, the specific method and operating steps are as follows:

Firstly, the processed SiC is scrubbed by detergent and then cleaned by the organic solvents to remove the stuck wax and other organic pollutants. The sequential order of the organic solvents to clean the SiC is n-hexane (C_6_H_14_), acetone (CH_3_COCH_3_), and anhydrous ethanol (C_2_H_5_OH), simultaneously vibrated by ultrasonic wave.

Secondly, the processed SiC is cleaned by the inorganic solvents to remove inorganic pollutants and strongly adsorptive pollutants by hydrogen peroxide (H_2_O_2_), hydrofluoric acid (HF), and hydrochloric acid (HCl), simultaneously vibrated by ultrasonic wave. 

Finally, the cleaned SiC is rinsed with deionized water and then dried off.

### 3.1. Effect of Ultrasonic on the Chemical Corrosion of Single Crystal SiC

**Figure 3:**
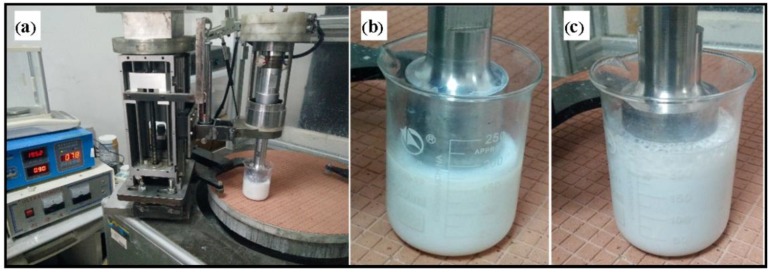
Physical picture of chemical corroding using CMP slurry: (**a**) Equipment for chemical corroding; (**b**) conventional chemical corroding; (**c**) ultrasonic chemical corroding.

**Figure 4:**
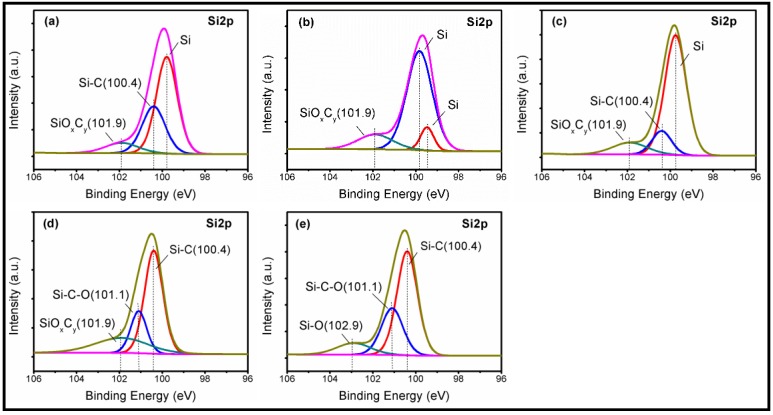
XPS Si2p spectra of Si-face 6H-SiC surfaces using CMP slurry: (**a**) before chemical corroding; (**b**,**c**) conventional chemical corroding (C-c’, U-c’); (**d**,**e**) ultrasonic chemical corroding (C-u’, U-u’).

As shown in Figure 3, the four combinational experiments of chemical corroding were conducted for 1 h using the CMP slurry in Table 3, respectively. Especially, it can be observed that large numbers of cavitation bubbles came up and the temperature of slurry rapidly rose in ultrasonic corroding. Before and after corroding, the Si-face SiC was measured by XPS respectively after cleaning in accordance with above step one and step three. The instrument for XPS measure was the ESCALAB-250Xi with the AI target. The size of Si-face SiC for XPS measure was smaller than 10 mm × 10 mm. Figure 4 shows the Si2p spectra of the Si-face 6H-SiC before and after chemical corroding using CMP slurry. Figure 4a is the Si-face SiC after lapping but before chemical corroding, where there exist three peaks due to Si, Si-C (100.4 eV) and SiOxCy (101.9 eV). The Si element and Si-C bond belong to Si-C bulk component while SiOxCy should originate from the frictional oxidation during lapping. Figure 4b,c are the Si2p spectra analysis results of conventional chemical corroding after conventional lapping and ultrasonic lapping, respectively. It can be seen that there are three peaks corresponding to Si, Si-C, and SiOxCy on the corroded surface. Compared with Figure 4a, the results cannot suggest an obvious oxidation. Figure 4e,f are the Si2p spectra analysis results of ultrasonic chemical corroding after conventional lapping and ultrasonic lapping, respectively. In Figure 4e, there are three main peaks due to Si-C (100.4 eV), Si-C-O (101.1 eV), and SiOxCy (101.9) [13,24,25] on the corroded surface, and in Figure 4f three main peaks corresponding to Si-C (100.4 eV), Si-C-O (101.1 eV), and Si-O (102.9 eV) also occur on the corroded surface. Compared with Figure 4a, the new presence of Si-C-O bond and Si-O bond suggest that the oxide layer formed on the Si-face surface after ultrasonic chemical corroding. The presence mechanism of Si-O bound can refer to the following Equations (2)–(5) [26,27]. The analysis results indicate that ultrasonic can contribute to driving the chemical corroding during CMP, which is due to the temperature rise and bubble movement, because when bubbles collapse in liquid, it results in an enormous concentration of energy from the conversion of the kinetic energy of liquid motion into heating of the contents of the bubble. The high local temperatures and pressures, combined with extraordinarily rapid cooling, provide a unique means for driving chemical reactions under extreme conditions [28].

(2) $$H_{2}O_{2}+{\mathrm{OH}}^{-}\overset{K_{\mathrm{eq}}}{↔}{\mathrm{HO}}_{2}{}^{-}+H_{2}O$$

(3) $$HO_{2}{}^{-}+H_{2}O_{2}\overset{k_{1}}{\to }H_{2}O+{\mathrm{OH}}^{-}+O_{2}(↑)$$

(4) $$\mathrm{SiC}+2HO_{2}{}^{-}+O_{2}\to {\mathrm{SiO}}_{2}+2{\mathrm{OH}}^{-}+CO_{2}(↑)$$

(5) $${\mathrm{SiO}}_{2}+2{\mathrm{OH}}^{-}\to {\mathrm{SiO}}_{3}^{2-}+H_{2}O$$

### 3.2. Effect of Ultrasonic on the CMP Performance of Single Crystal SiC

**Figure 5:**
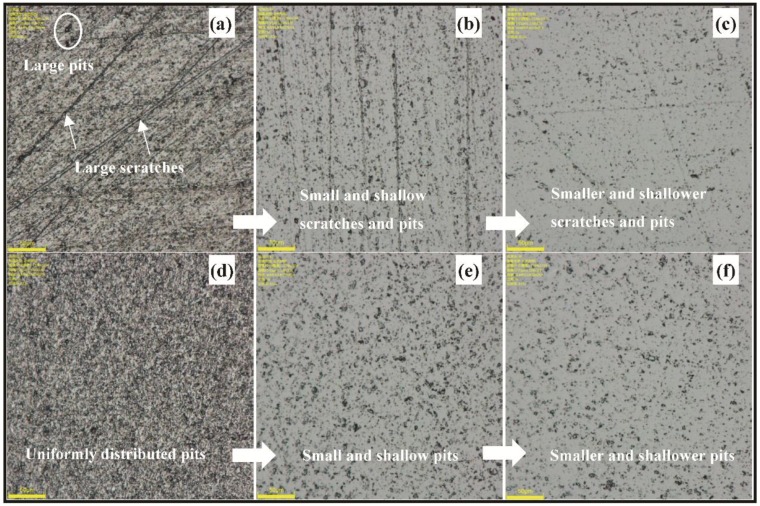
Microscope images (832×) of Si-face SiC processed by the designed comparative experiments: (**a**) conventional lapping; (**b**) conventional CMP after conventional lapping(C-c); (**c**) ultrasonic CMP after conventional lapping (C-u); (**d**) ultrasonic lapping; (**e**) conventional CMP after ultrasonic lapping (U-c); (**f**) ultrasonic CMP after ultrasonic lapping (U-u).

Figure 5 is the microscope image (832×) of Si-face SiC processed by the designed comparative experiments. Figure 5a,d are the surface texture of conventional lapping and ultrasonic lapping, respectively, both the two kinds of lapping experiments are conducted by a two-step method under the pressure of 20 KPa, rotating speed of 40 rpm, and slurry supplying rate of 10 mL/min. The first step is using the diamond abrasives with the average diameter of 4 μm to conduct the lapping experiment for 5 min, and the second step is using Al_2_O_3_ abrasives sized W1.5 to conduct the lapping experiment for 5 min. Comparing conventional lapping with ultrasonic lapping, it can be obviously found that the surface is characterized with random large scratches and large pits in the conventional lapping while the surface is characterized with the uniformly distributed large pits in the ultrasonic lapping. It can be inferred that in ultrasonic lapping the severe mechanical damages from abrasive scratching are reduced probably because the normal force of abrasive particle is decreased, and the uniformly distributed large pits should come from the ultrasonic impacting and dispersion. Figure 5b,c are conventional CMP and ultrasonic CMP after conventional lapping, respectively. Compared with Figure 5b, the surface gets better improvement in Figure 5c, whose scratches and pits are both shallower and less. The result indicates that more defects including severe scratches and pits left in the former process are removed because of a higher MRR than conventional CMP. Similarly, it also can be seen that the number of pits in Figure 5f is less compared with Figure 5e. Based on the two observations, it can be confirmed that ultrasonic is helpful to material removal in CMP, thus improving the surface quality faster.

**Figure 6:**
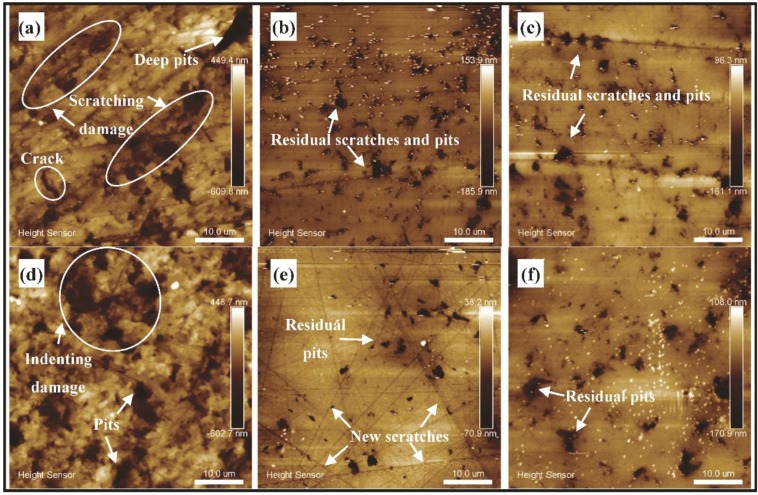
The variation of Si-face SiC surface morphology processed by different ways through atomic force microscopy (AFM) measurement (50 × 50 μm^2^ area): (**a**) conventional lapping; (**b**) conventional CMP after conventional lapping(C-c); (**c**) ultrasonic CMP after conventional lapping (C-u); (**d**) ultrasonic lapping; (**e**) conventional CMP after ultrasonic lapping (U-c); (**f**) ultrasonic CMP after ultrasonic lapping (U-u).

Figure 6 is the result of surface generation corresponding to Figure 5 on a smaller scale, which is measured through AFM with the area of 50 × 50 μm^2^. Figure 6a,d are the results of conventional lapping and ultrasonic lapping separately, Figure 6b,c are the results of conventional CMP and ultrasonic CMP after conventional lapping, Figure 6e,f are the results of conventional CMP and ultrasonic CMP after ultrasonic lapping. The results show the mechanism of surface generation and the characteristic of surface damage. In conventional lapping, the material removal mainly originates from the abrasive scratching, which leads to obvious scratching damages including scratches, crack and brittle fracture. The other defect is the deep pit, which should be produced by the abrasive rolling. In contrast with conventional lapping, few scratches can be seen but deep pits and indenting damage are the main defects in ultrasonic lapping, which demonstrates that abrasive impacting and rolling make the main contribution to material removal. There are residual scratches and pits in CMP with and without ultrasonic vibration after conventional lapping. Conventional CMP after ultrasonic lapping produces new scratches compared with ultrasonic CMP after ultrasonic lapping, but both processes have residual pits. The ultrasonic lapping and ultrasonic CMP suggest that ultrasonic can reduce the abrasive scratching but increase the abrasive impacting. The residual scratches and pits after CMP demonstrate that high MRR with little damage needs a balance between lapping and CMP. Additionally, the weight loss of a single crystal SiC wafer before and after processing are listed in Table 4. The corresponding MRRs are calculated according to Equation (1) after the surface oxides produced in CMP are removed by dipping in HF for 2 h, where the chemical reaction mechanism is shown in Equation (6). The surface roughness of Ra, Rq (namely root mean square, RMS), Rmax (namely peak-to–valley value, PV) after conventional lapping are 96.8 nm, 133 nm, 1.651 μm and the corresponding values after ultrasonic lapping are 116 nm, 149 nm, and 1.584 μm. The MRRs of the two lapping are 1.48 μm/min and 1.62 μm/min, respectively. The results show that ultrasonic lapping has a higher MRR and lower PV value but the higher Ra and Rq, which indicates that ultrasonic can help to decrease the depth of damaged layer and improve efficiency, but does not decrease the average roughness. The surface roughness of Ra, Rq, and Rmax in Figure 6b,c are respectively 21.0 nm, 37.3 nm, and 1.115 μm, and 20.2 nm, 32 nm, and 0.771 μm. The corresponding values in Figure 6e,f are respectively 8.82 nm, 15.1 nm, and 0.477 μm, and 20.2 nm, 31.4 nm, and 0.474 μm. The results show that the PV value of ultrasonic CMP is smaller than conventional CMP. Both the results of ultrasonic lapping and ultrasonic CMP conform that ultrasonic vibration can decrease two-body abrasion [21]. Moreover, the surface improvement of CMP after ultrasonic lapping are both better than CMP after conventional lapping, which means that ultrasonic lapping is beneficial for the following CMP to get a better surface probably because of the lower PV value and higher MRR.

(6) $${\mathrm{SiO}}_{2}+4\mathrm{HF}\to {\mathrm{SiF}}_{4}(↑)+2H_{2}O$$

**Figure 7:**
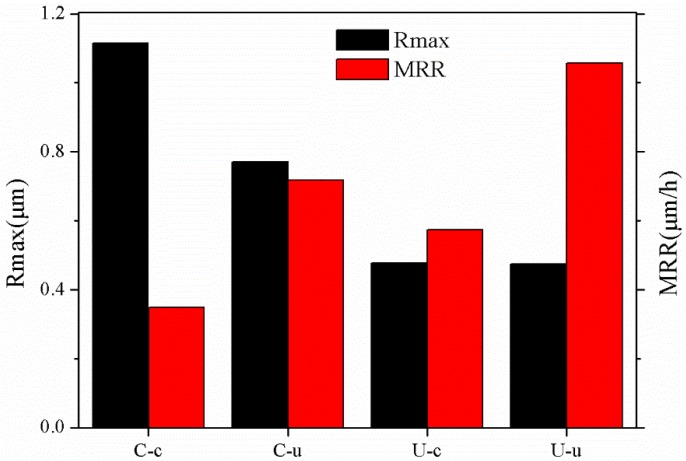
The peak-to-valley value of roughness (Rmax) and material removal rate (MRR) of CMP after lapping.

Based on the weight change in Table 4, the MRRs of C-c, C-u, U-c, and U-u can be calculated as 0.347 μm/h, 0.717 μm/h, 0.574 μm/h, and 1.057 μm/h, respectively, which are all higher than the MRR of 100 nm/h in the preceding reports. Combined with Figure 7, it cannot be difficult to find that the MRRs of ultrasonic CMP (C-u and U-u) are both higher than corresponding conventional CMP (C-c and U-c) and the MRRs of CMP after ultrasonic lapping (U-c and U-u) are higher than corresponding conventional lapping (C-c and C-u), moreover ultrasonic CMP combined with ultrasonic lapping (U-u) achieves the highest MRR of 1.057 μm/h with the lowest PV of 0.474 μm. The results indicate that ultrasonic lapping is beneficial to increase the MRR of CMP, and ultrasonic CMP can also achieve higher MRR and lower PV compared with conventional CMP. Therefore it is demonstrated that ultrasonic CMP combined with ultrasonic lapping for the SiC is a feasible method to increase the efficiency and surface quality.

**Figure 8:**
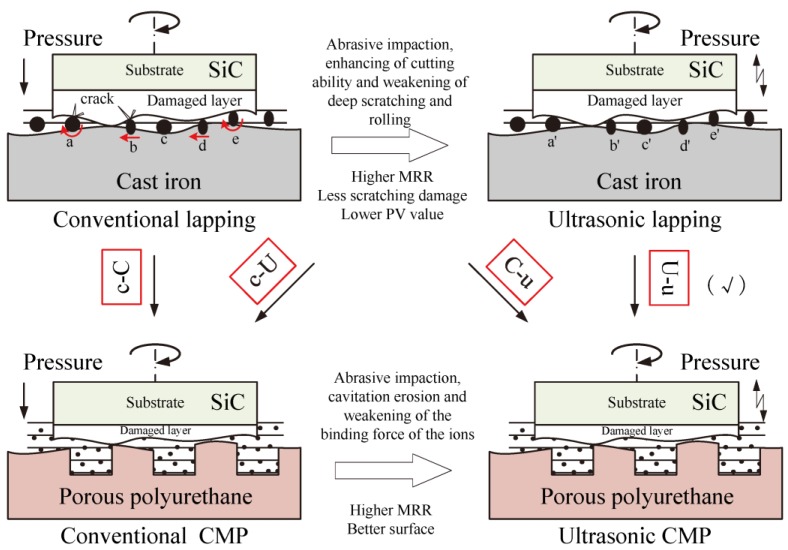
The material removal mechanism of CMP and lapping for SiC and its varied action under ultrasonic. The abrasive action in conventional lapping includes brittle three-body abrasion (**a**), brittle two-body abrasion (**b**), no contact (**c**), ductile two-body abrasion (**d**), and ductile three-body abrasion (**e**).

Figure 8 shows the material removal mechanism of CMP and lapping for SiC and its varied action under ultrasonic. As shown in conventional lapping, the abrasive action includes (a) brittle three-body abrasion; (b) brittle two-body abrasion; (c) no contact; (d) ductile two-body abrasion; and (e) ductile three-body abrasion. In ultrasonic lapping, the corresponding abrasive action of (a’~e’) has a great change. For the brittle three-body abrasion and brittle two-body abrasion, ultrasonic weakens the deep scratching and rolling action which can reduce the subsurface damage. For the ductile three-body abrasion and ductile two-body abrasion, the ultrasonic can increase the fracture toughness which is beneficial for achieving ductile removal and the subsurface damage is less serious [29,30]. Furthermore, the abrasive impaction can enhance the cutting ability to increase the material removal [29]. Chemical mechanical polishing is used to smooth the surface by removing the residual damages from lapping, and the material removal of conventional CMP mainly originates from the synergistic action of chemical corrosion and abrasive mechanical action. In ultrasonic CMP, the enhanced chemical corrosion, enhanced contact force between the contact particles and the machining surface, and the impact of the suspending particles to the machining surface contribute to material removal, by simultaneously weakening the binding force of the ions that makes the SiC surface material easy to be removed [17,19]. Besides, the ultrasonic can effectively reduce the wear coefficients [21], which can convert the severely damaged action of two-body abrasion to the less damaged action of three-body abrasion thereby leading to little new scratches compared to the conventional CMP. Therefore, the ultrasonic chemical mechanical polishing of a single crystal SiC wafer combined with ultrasonic lapping is a feasible method to acquire a higher MRR with a less damaged surface.

## 4. Conclusions

In this study, the novel sequent processing method of ultrasonic CMP combined with ultrasonic lapping was employed to finish the Si-face SiC. And an ultrasonic assisted machining system was designed and manufactured. After the comparative experiments, it can be found that the combined ultrasonic CMP with ultrasonic lapping achieved the highest MRR of 1.057 μm/h and lowest PV value of 0.474 μm. The reason for this result can be implied by the following indications. After ultrasonic corroding, the presence of a Si-C-O bond and Si-O bond indicated that ultrasonic can contribute to driving the chemical corroding of Si-face SiC in CMP slurry. After ultrasonic lapping, the severe mechanical damage from abrasive scratching was reduced probably because the normal force of abrasive particle decreased, and the uniformly distributed large pits should come from the ultrasonic impacting and dispersion. Abrasive impacting and rolling made the main contribution to material removal in ultrasonic lapping. Besides, ultrasonic lapping has a higher MRR and lower PV value but the higher Ra and Rq, which indicates that ultrasonic can help to decrease the depth of damaged layer with a higher efficiency but does not decrease the average roughness. What’s more, ultrasonic lapping following CMP is beneficial to get a better surface, probably because of the lower PV value and higher MRR. After ultrasonic CMP, more defects including severe scratches and pits left in the former process are removed because ultrasonic CMP can also achieve higher MRR and lower PV value compared with conventional CMP. Both ultrasonic lapping and ultrasonic CMP can decrease two-body abrasion and have a lower PV value. Therefore, it is demonstrated that ultrasonic CMP combined with ultrasonic lapping for the SiC is a feasible method to increase the efficiency and surface quality. Moreover, this sequent ultrasonic assisted process can be suitable for the hard-brittle and strong chemical inertial materials like SiC. But the residual scratches and pits after CMP demonstrate that high MRR with little damage need a balance between lapping and CMP. 

## Figures and Tables

**Table 1:** Experimental schemes for corroding and CMP after lapping.

Corroding	CMP
Conventional corroding After conventional lapping (C-c’)	Conventional CMP After conventional lapping (C-c)
Ultrasonic corroding After conventional lapping (C-u’)	Ultrasonic CMP After conventional lapping (C-u)
Conventional corroding After ultrasonic lapping (U-c’)	Conventional CMP After ultrasonic lapping (U-c)
Ultrasonic corroding After ultrasonic lapping (U-u’)	Ultrasonic CMP After ultrasonic lapping (U-u)

**Table 2:** Experimental conditions for lapping.

Category	Parameters
Specimen	6H-SiC wafer: 2 inch × (440 ± 25) μm (from the Tankeblue semiconductor, Beijing, China)
Mechanical action	Abrasive: diamond (4 μm/4 wt%), Al_2_O_3_ (W1.5/6 wt%) Slurry supplying rate: 10 mL/min Lapping disc: cast iron (φ500 mm) Revolution speed of disc: 40 rpm Pressure: 20 KPa
Ultrasonic vibration	Maximum power (Pmax): 2 KW Setting power: 70% × Pmax (*f* = 19.54 KHz, *A* = 2 μm)
Time	5 min diamond + 5 min Al_2_O_3_

**Table 3:** Experimental conditions for CMP.

Category	Parameters
Ultrasonic vibration	Maximum power (Pmax): 2 KW Setting power: 70% × Pmax (*f* = 19.54 KHz, *A* = 2 μm)
Mechanical action	Platen rotation speed: 40 rpm (0.6–1 m/s) Download pressure: 60 KPa Pad: Porous Polyurethane (shenyang kejing, China)
Slurry	Type: colloidal silica Supplying rate: 25 mL/min Abrasive size/mass fraction: Average 100 nm/20 wt% Oxidant and catalyst: 6 wt% H_2_O_2_ and 0.6 wt% alkali KOH
Time	1 h

**Table 4:** Weight loss before and after processing.

Experiments	Before (g)	After (g)	Weight Change (g)
Conventional lapping	2.8612	2.7635	0.0977
Ultrasonic lapping	2.7750	2.6645	0.1105
C-c	2.7640	2.7617	0.0023
C-u	2.7539	2.7492	0.0047
U-c	2.6821	2.6783	0.0038
U-u	2.7645	2.7575	0.0070
